# A Comparison of the Effectiveness of Neurodynamic Sliding Technique and Self-Myofascial Release Technique for Reducing Hamstring Tightness in Healthy Individuals: A Prospective Study

**DOI:** 10.7759/cureus.40613

**Published:** 2023-06-18

**Authors:** Amreen Afsarpatel Shaikh, Maliha Fatima Quraishi, Tajuddin Chitapure, Priyanka Abhay Joshi, Saba Afsar Shaikh, Nikita Nandgaonkar, Komal Sable

**Affiliations:** 1 Clinical Physiotherapy, Musculoskeletal Department, MGM School of Physiotherapy, Mahatma Gandhi Mission Institute of Health Sciences (MGMIHS), Aurangabad, IND; 2 Physiotherapy, Musculoskeletal Department, MGM School of Physiotherapy, Mahatma Gandhi Mission Institute of Health Sciences (MGMIHS), Aurangabad, IND; 3 Musculoskeletal Physiotherapy, Musculoskeletal Department, MGM School of Physiotherapy, Mahatma Gandhi Mission Institute of Health Sciences (MGMIHS), Aurangabad, IND

**Keywords:** healthy individual, active knee extension, neurodynamic sliding technique, myofascial release, hamstring tightness

## Abstract

Background: Hamstring tightness is prevalent among college-going students aged 18-25 years, leading to an increased risk of recurrent injury, reduced athletic performance, post-exercise soreness, and decreased coordination. Myofascial release and neurodynamic sliding technique are two interventions used to alleviate this issue. Myofascial release is a concept that involves pain originating from the muscle and fascia. The neurodynamic sliding technique is a method of producing sliding movement of neural structures relative to their mechanical interfaces.

Methods: This study involved 70 individuals with hamstring tightness who met the inclusion and exclusion criteria. Participants were assigned to Group A or Group B using a convenient sampling method. Group A received neurodynamic sliding technique treatment, while Group B received a self-myofascial release. Both interventions were administered for two months. The outcome measures used in this study were active knee extension and lower extremity functional scale, which were evaluated before and after the intervention.

Results and implications: Within-group comparisons indicated that both Group A and Group B showed significant improvements in hamstring flexibility. Between-group comparisons of active knee extension (AKE) and lower extremity functional scale (LEFS) immediately after the intervention showed statistically significant results. These findings suggest that both the neurodynamic sliding technique and self-myofascial release are effective in improving hamstring flexibility. This study has implications for clinical practice, as both interventions may be used to address hamstring tightness.

Conclusion: Our study found that both the neurodynamic sliding technique and self-myofascial release can improve hamstring flexibility. However, the neurodynamic sliding technique was found to be more effective than self-myofascial release. Further research is necessary to determine the optimal protocol for these interventions and their effectiveness in clinical populations with hamstring tightness or injury.

## Introduction

Injuries to the hamstrings are a common phenomenon observed during high-intensity physical activities. To understand the underlying causes that contribute to the frequent occurrence of such injuries, extensive research has been conducted [1]. The majority of these investigations have revealed that tightness of the hamstring muscle group exacerbates lower extremity injuries [2]. While it is widely acknowledged among health professionals and researchers that hamstring flexibility plays a crucial role in injury prevention, there is a lack of consensus on the optimal approach for hamstring stretching [3]. During ambulation, the hamstring causes a reduction in knee extension to protect the hip and knee joints while also providing dynamic stability. The interaction of the hamstring with the knee allows for proper knee mobility and stability [4]. The hamstring is a typical example of a muscle group that tends to shorten. Muscle tightness occurs due to a reduction in the muscle's capacity to deform, resulting in a decrease in the range of motion in which it operates. Hamstring tightness results in an inability to achieve more than 160 degrees of knee extension when the hip is at 90 degrees of flexion [5,6].

Hamstring tightness is remarkably prevalent among adolescents aged 18-25 [7]. Hamstring muscles are frequently associated with movement dysfunction at the lumbar spine and pelvis and have been linked to low back pain and gait abnormalities. Limited flexibility resulting from hamstring tightness triggers neuro-musculoskeletal symptoms, thereby decreasing strength, stability, endurance, and other related factors. Hamstring tightness is closely linked to a posterior rotation of the pelvis in standing, primarily due to the attachment of the hamstring muscle on the ischial tuberosity. Tightness in the hamstring muscle group leads to a posterior pelvic tilt, which decreases lumbar lordosis, ultimately leading to low back pain [8,9]. Only a limited number of studies have examined the effects of active release technique and dynamic stretching on athletic performance. The majority of static stretching research concluded that it was harmful to hamstring muscle performance. According to the research conducted by Church et al. (2001) [10], incorporating the active release approach during stretching has been found to decrease the likelihood of anterior cruciate ligament (ACL) injury.

Myofascial release is a concept of unique, distinct pain that originates in both the muscle and fascia. Pain is described as deep within the target tissue as being sharp, burning, dull, boring like toothache, heavy, or squeezing [11]. Myofascial release (MFR) is a commonly employed manual technique aimed at promoting uninterrupted tissue stretching and enhancing the flexibility of soft tissues through compression. It helps in restoring restricted fascia and improving muscle length by utilizing compression to facilitate continuous stretching of the relevant tissues [12]. MFR is focused on the application of gradual, continuous pressure to restricted fascial layers for 120 to 300 seconds [13]. Self-myofascial release (self-MFR) is a modality within the domain of myofascial release, wherein individuals utilize tools instead of relying on the assistance of a therapist. Self-MFR is a low-cost, easily accessible way for people to relieve muscle and fascia pain while maintaining flexibility [14]. Foam rollers and roller massage are two of the most used tools for self-MFR. Constantly, self-MFR improves flexibility, lowers delayed onset muscle soreness, modifies arterial and hemangioendothelioma function, which is an abnormal endothelial cell tumor affecting blood vessel structure and function, and regulates the autonomic nervous system [15]. Self-MFR techniques have gained immense popularity in fitness and rehabilitation societies for preventing and treating pathologies. One such technique is the neurodynamic sliding technique (NDST), which involves producing sliding movements of neural structures relative to their mechanical interfaces. This method applies pressure on the targeted nerve proximally through joint movements, releases distal tension, and then reverses the sequence. Neurodynamics involves the interplay between nervous system mechanics and physiology. Any alterations in neurodynamics of the lower extremities could potentially affect the length of the entire body and cause changes in the perception of stretch or pain [16].

The application of motion or stretching may lead to changes in neurodynamics and a shift in perception, which could help explain the observed increase in flexibility. Additionally, the mechano-sensitivity of the neural structures in the posterior limb, thigh, buttock, and vertebral canal may also play a role in determining the hamstring muscle's flexibility [17]. Protective muscle contraction of the hamstring muscles discovered in the presence of neural mechano-sensitivity can cause hamstring tightness and thus predispose the muscle to subsequent stress injury. Neurodynamic sliding interventions are believed to reduce neural mechano-sensitivity in the incorporation of these interventions in hamstring flexibility management is shown to be useful [18]. Neurodynamic stretching is a manual form of stretching in which force is applied to nerve structures via posture and multi-joint movement, intending to cause neural structures to slide relative to their surrounding tissues [19].

Neurodynamics is considered to reduce neural mechanosensitive and can be a useful therapy for hamstring flexibility management [16]. Neurodynamic changes in nerve system mobility brought about by movement and stretching could alter such sensations. NDST therapies to reduce neural mechanosensitive; this intervention may help with hamstring flexibility. Alterations in neurodynamics affect the sciatic, tibial, and common fibular nerves which could cause decreased hamstring flexibility as shown by limited range in the passive straight leg raise test (SLR) [16].

The research question addressed whether the NDST or self-MFR was more beneficial in terms of improving the parameters of the Active Knee Extension (AKE) test and the Lower Extremity Functional Scale (LEFS) in individuals with hamstring tightness. The main objective of this study was to investigate whether the NDST yielded superior outcomes in enhancing hamstring flexibility when compared to MFR. The primary objectives included investigating the effect of the NDST on hamstring tightness. The secondary objectives were twofold: first, to investigate the effect of self-MFR on hamstring tightness, and second, to compare the effectiveness of NDST and self-MFR in reducing hamstring tightness. Although there were numerous studies on hamstring tightness in India, this issue was prevalent among healthy individuals, and the effectiveness of NDST and MFR had been studied independently. Therefore, this study aimed to ascertain which technique was more effective and cost-efficient in reducing hamstring tightness.

## Materials and methods

The study was conducted at the Musculoskeletal Outpatient Department of MGM School of Physiotherapy in N-6 Cidco, Aurangabad. The study design was a prospective comparative study, and sample selection was done using the coin method with convenient sampling. The study population consisted of healthy individuals, with a sample size of 70, calculated statistically depending on the dropout rate. The study duration was 12 months.

Inclusion criteria included individuals aged 18-25 years, of both genders, with normal joint mobility and AKE less than 125°. Exclusion criteria included individuals with regular sports participation, low joint mobility, hamstring strain, metabolic disease, hamstring tear, neurological diseases, and rheumatoid arthritis.

The outcome measure consisted of the AKE test and the LEFS. AKE involved measuring the angle of knee flexion while supine, with the hips, flexed at 90 degrees and knees flexed, using a full circle goniometer. LEFS was a self-report questionnaire that assessed the patient's ability to perform 20 different everyday activities.

Procedure

This study was conducted at the Mahatma Gandhi Mission School of Physiotherapy, Aurangabad. Healthy Individuals with hamstring tightness were recruited from the same institute where the study was conducted. The study procedure was initiated after the approval of MGM’s Ethics Committee for research on human subjects (MGM-ECRHS/2019/92). Consent was taken from all participants before enrolling them in the study.

All the participants who had previously been diagnosed with hamstring tightness and who met the inclusion criteria were invited to participate in the study after being briefed about the study and intervention. Written informed consent was obtained from all participants, and they were divided into two groups.

Group A received NDST for hamstring tightness and warm-up exercises, while Group B received self-MRT for hamstring tightness and warm-up exercises. Both groups were evaluated using outcome measures, including AKE and LEFS, before and after a two-month treatment period.

After completing the initial assessment prior to treatment, the participants were positioned in a seated position to undergo the NDST with their trunk in thoracic flexion and performed alternating movements of knee extension, ankle dorsiflexion with cervical extension, and knee flexion, ankle plantar flexion with cervical flexion for approximately 60 seconds for five times. Self-MFR involved participants being in a long-sitting position on an even surface, placing their arms backward, and loading body weight on the palms. A foam roller was placed under the hamstrings, and pressure was applied by slowly moving back and forth from the ischial tuberosity to the popliteus for four minutes daily for two months.

## Results

Table 1 displays the results of a Shapiro-Wilk test that was conducted to check for normality. Out of AKE Right, AKE Left, LEFS Right, and LEFS Left, none of the variables showed a non-significant outcome for either Group A or Group B. Therefore, non-parametric tests were used for data analysis in the following sections.

**Table 1:** Normality Test Using Shapiro-Wilk Test AKE: Active Knee Extension, LEFS: Lower Extremity Functional Scale

Variable	Time frame	Group A		Group B	
		z-value	p-value	z-value	p-value
AKE Right	Pre	0.807	0.001	0.846	0.001
	Post	0.914	0.010	0.920	0.014
AKE Left	Pre	0.820	0.001	0.925	0.019
	Post	0.914	0.010	0.862	0.001
LEFS Right	Pre	0.956	0.169	0.943	0.071
	Post	0.929	0.027	0.954	0.147
LEFS Left	Pre	0.914	0.010	0.947	0.093
	Post	0.957	0.183	0.925	0.020

Table 2 presents a crosstabulation of group and gender. The chi-square value of 0.059 and p-value of 0.808 indicated that there was no significant association between group and gender.

**Table 2:** Group * Gender Crosstabulation

		Gender		Total	Chi square	p-value
		Male	Female			
Group	Grp-A	15	20	35	0.059	0.808
	Grp-B	14	21	35		
Total		29	41	70		

Table 3 shows the descriptive statistics for age, weight, height, and BMI for both Group A and Group B. There were no significant differences between the groups in terms of these variables.

**Table 3:** Group Descriptive Statistics for Age, Weight, Height, and BMI

Variable	Group	Mean	SD	t-test	p-value
Age	Grp-A	22.74	1.62	0.724	0.472
	Grp-B	22.46	1.69		
Weight	Grp-A	55.09	5.43	0.072	0.943
	Grp-B	55.00	4.52		
Height	Grp-A	165.37	8.82	0.525	0.601
	Grp-B	164.20	9.83		
BMI	Grp-A	20.22	2.20	1.083	0.283
	Grp-B	25.43	28.42		

Table 4 presents the results of a within-group analysis using a paired Wilcoxon test for Group A. AKE Right, AKE Left, LEFS Right, and LEFS Left mean values showed changes post-treatment, and higher mean values were recorded for the post-treatment outcome. The effect size, measured by Cohen's D, was high for all variables. The p-values for all variables were less than the 5% significance level, indicating a significant and reliable difference between pre- and post-treatment values. Therefore, the results justified the improvements in health outcomes post-intervention.

**Table 4:** Within-Group Pre- and Post-Paired Wilcoxon Test for Group A With * Significant at 5% Level AKE: Active Knee Extension, LEFS: Lower Extremity Functional Scale

Variable	Mean Ranks		Sum of Ranks		Pre		Post		Effect size	z-value	p-value
	Negative	Positive	Negative	Positive	Mean	SD	Mean	SD			
AKE Right	0.00	18	0.00	630	117.80	3.72	126.77	2.26	3.08	5.174	0.001*
AKE Left	0.00	18	0.00	630	118.57	3.00	127.49	1.90	3.26	5.173	0.001*
LEFS Right	0.00	18	0.00	630	15.49	5.50	71.34	4.70	8.51	5.163	0.001*
LEFS Left	0.00	18	0.00	630	16.63	3.98	69.83	3.54	9.66	5.162	0.001*

Upon conducting a within-group analysis using a paired test, it was observed that there were changes in the mean values for AKE Right, AKE Left, LEFS Right, and LEFS Left post-treatment, with higher mean values recorded for the post-treatment outcome. The standard deviation showed consistency with the post-treatment value, which was lower than the pre-treatment value. The effect size, as measured by Cohen's D, was high for all variables, with values of 3.08, 3.26, 8.51, and 9.66 for AKE Right, AKE Left, LEFS Right, and LEFS Left, respectively. These values are very high according to standard reference parameters.

Based on the results of the test analysis at a 5% significance level, there was a significant and reliable difference between the pre- and post-treatment values, with p-values less than the 5% significance level (i.e., 0.001<0.05) in the study. Therefore, the improvements in health outcomes post-intervention are justified.

The findings of the within-group pre- and post-treatment paired Wilcoxon test for Group B are presented in Table 5. The table includes the mean ranks, sum of ranks, and mean and standard deviation values for the negative and positive scores of the variables AKE Right, AKE Left, LEFS Right, and LEFS Left. The effect size is measured by Cohen's D value, and the statistical significance of the results is determined by the z-value and p-value. The results demonstrate that there were significant improvements in all four variables post-treatment, as indicated by the high effect sizes and p-values of less than 0.05, suggesting a statistically reliable difference between pre- and post-treatment values.

**Table 5:** Within-Group Pre- and Post-Paired Wilcoxon Test for Group B With * Significant at 5% Level AKE: Active Knee Extension, LEFS: Lower Extremity Functional Scale

Variable	Mean Ranks		Sum of Ranks		Pre		Post		Effect size	z-value	p-value
	Negative	Positive	Negative	Positive	Mean	SD	Mean	SD			
AKE Right	0.00	18	0.00	630	118.66	3.46	125.29	1.58	2.11	5.180	0.001*
AKE Left	0.00	18	0.00	630	117.97	3.24	122.91	1.96	2.29	5.176	0.001*
LEFS Right	0.00	18	0.00	630	13.06	5.19	73.00	2.85	9.38	5.162	0.001*
LEFS Left	0.00	18	0.00	630	12.40	4.10	70.37	2.72	10.90	5.163	0.001*

The analysis of within-group paired tests revealed changes in mean values for AKE Right, AKE Left, LEFS Right, and LEFS Left post-treatment. Higher mean values were recorded for post-treatment outcomes, and the standard deviation indicated consistency with post-treatment values, which were lower than pre-treatment values. The effect size or Cohen’s D showed a high value of 2.11 for AKE Right, 2.29 for AKE Left, 9.38 for LEFS Right, and 10.90 for LEFS Left, as per the standard parameters of reference. Based on the statistical test analysis at a 5% significance level, there was a significant, reliable difference between pre- and post-treatment values, with p-values less than the 5% significance level (i.e., 0.001<0.05) in the study, which supports the improvement in health outcomes post-intervention.

Table 6 presents the results of the Mann-Whitney test for between-group statistics. The table includes variables such as AKE Right, AKE Left, LEFS Right, and LEFS Left with their respective mean rank, the sum of ranks, z-value, and p-value for each group and time frame. The results indicate that there is a significant difference between groups for AKE Right post, AKE Left post, and LEFS Left pre, as the p-value is less than 5% (0.001<0.05). Group A demonstrates higher mean ranks and the sum of ranks, indicating that it performs better than Group B.

**Table 6:** Between-Group Statistics Mann-Whitney Test AKE: Active Knee Extension, LEFS: Lower Extremity Functional Scale

Variable	Time Frame	Group	Mean Rank	Sum of Ranks	z-value	p-value
AKE Right	Pre	Group A	32.21	1127.50	1.376	0.169
		Group B	38.79	1357.50		
	Post	Group A	42.90	1501.50	3.088	0.002*
		Group B	28.10	983.50		
AKE Left	Pre	Group A	37.61	1316.50	0.883	0.377
		Group B	33.39	1168.50		
	Post	Group A	51.70	1809.50	6.699	0.001*
		Group B	19.30	675.50		
LEFS RIGHT	Pre	Group A	39.87	1395.50	1.802	0.072
		Group B	31.13	1089.50		
	Post	Group A	32.49	1137.00	1.247	0.213
		Group B	38.51	1348.00		
LEFS LEFT	Pre	Group A	45.07	1577.50	3.952	0.001*
		Group B	25.93	907.50		
	Post	Group A	33.63	1177.00	0.775	0.438
		Group B	37.37	1308.00		

## Discussion

The aim of our study was to compare the efficacy of the self-MFR technique and NDST in reducing hamstring tightness in healthy individuals. Our study found that both interventions were effective in reducing hamstring tightness, but Group A (NDST) showed a greater improvement compared to Group B (self-MFR technique). The LEFS and AKE for both right and left sides were used as outcome measures, which were recorded before and after treatment and at the completion of two months. Hamstring tightness is becoming increasingly common among healthy individuals, particularly among those with sedentary lifestyles. The mechanism of hamstring tightness in sedentary individuals involves several factors. Prolonged sitting or a sedentary lifestyle can lead to a lack of regular physical activity and muscle disuse. This, in turn, can result in muscle imbalances and decreased flexibility in the hamstring muscles. Additionally, prolonged sitting can cause adaptive changes in the musculoskeletal system, leading to shortened and tight hamstring muscles. Poor posture and inadequate stretching or mobility exercises can further contribute to hamstring tightness in sedentary people. Previous research by Khalil et al. in 2022 has shown that sedentary lifestyles are common among healthy individuals and can hinder activities of daily living, potentially leading to further health issues [20]. Furthermore, our study included a higher proportion of female participants with hamstring tightness, which is consistent with prior research indicating that females exhibit a higher prevalence of hamstring tightness due to a lack of regular flexibility exercises or improper physical activities [21]. Additionally, research by Singh et al. in 2015 demonstrated that working women are more likely to experience work-related musculoskeletal disorders, many of which are linked to tight muscles, particularly the hamstrings [22].

According to Subburam 2018, the NDST is more effective because it targets both muscles and nerves, improving hamstring flexibility by reducing neural mechanosensitivity. The sliding technique involves combining movements, which lengthens the nerve bed and activates the muscle at one joint while shortening the nerve bed at an adjacent joint [23]. Castellote-Caballero et al. in 2014 compared the NDST with static muscle stretching, finding that a single neurodynamic intervention resulted in a higher instantaneous increase in passive SLR range of motion. The authors suggest that this result may be attributed to Weppler and Magnusson's "sensory theory," which suggests that perceptions of stretch and pain (sensation) have a greater impact on muscle flexibility and its response to rapid stretching than biomechanical effects on the muscle tissue itself [16].

De Ridder and colleagues in 2020 demonstrated that after a six-week intervention, neurodynamic sliders offer linear sciatic nerve excursion, leading to increased hamstring flexibility as compared to static stretching. This mechanism could be attributed to the reduction of neural mechanosensitivity and the enhancement of neural tissue viscoelasticity, thereby promoting hamstring mobility [24]. Similarly, the self-MFR group has also shown a significant improvement in hamstring flexibility. Sakhalkar and colleagues in 2022 suggested that the effectiveness of self-MFR could be attributed to the release of the elastocollagenous complex's tissues from stress through increased soft tissue elasticity. This results in improved metabolism and a better state of health by reducing the density and viscosity of the matrix [25]. Additionally, Jihye Jung et al., 2017, saw the immediate effect of self-MFR on hamstring flexibility. The immediate effect is due to their research suggesting that people who need to increase muscle flexibility might find the indirect application based on the Anatomy Trains to be useful. Furthermore, self-MFR can be done anywhere at any time to quickly and effectively reduce myofascial pain while maintaining flexibility [15]. Krishna and colleagues, 2021, compared the immediate effect of neural mobilization and MFR of the suboccipital muscle on hamstring length and concluded that neurodynamics has a better immediate effect in increasing hamstring length. This is because tension applied to the nervous system while applying neurodynamics results in an extension and movement of the sciatic nerve, resulting in increased flexibility. Myofascial release of the suboccipital muscle could also increase the flexibility of the hamstring due to the relaxation of the superficial backline through the relaxation of suboccipital muscles [26]. However, no study has compared the effectiveness of neurodynamic sliding and self-MFR. Our study has found that neurodynamic sliding is more effective than self-MFR in increasing hamstring flexibility, based on the results obtained in healthy individuals.

## Conclusions

In conclusion, our study found that both the NDST and self-MFR are effective in improving hamstring flexibility, but the NDST showed a greater improvement compared to the self-MFR technique. However, further research is needed to determine the optimal protocol for these interventions and whether they are effective in clinical populations with hamstring tightness or injury.
